# A creep in the vancomycin minimum inhibitory concentration for *Staphylococcus aureus *in a tertiary care hospital in India

**DOI:** 10.1186/cc9652

**Published:** 2011-03-11

**Authors:** A Bhakta, M Bhattacharyya, S Todi

**Affiliations:** 1 AMRI Hospitals, Kolkata, India

## Introduction

Vancomycin minimum inhibitory concentration (MIC) creep has been observed in studies from western countries. *Staphylococcus aureus *strains with increased MIC to vancomycin are associated with worse outcomes compared with more susceptible strains. Recognition of this phenomenon - the development of reduced susceptibility to vancomycin, and the subsequent glycopeptide MIC creep - is important, since it may be a precursor to heterogeneous vancomycin intermediate *S. aureus *(hVISA) and VISA.

## Methods

In a study carried out in a tertiary care hospital in India, 176 clinically significant Gram-positive bacterial isolates were collected from January 2009 to October 2009. MICs were determined for vancomycin, teicoplanin, linezolid, daptomycin and cefoxitin (to screen for methicillin-resistant *S. aureus*) using the E strips.

## Results

Out of 176 isolates, 72 were MSSA, 16 MRSA, 68 Enterococcus spp. and 20 coagulase-negative staphylococcus. No VISA or VRSA was detected. Sixteen MRSA and 12 MSSA isolates have MIC 2. The MIC50 values of MRSA and MSSA were 1.5 and 1, respectively. The MIC90 values of MRSA and MSSA were 2 and 1.5, respectively. A total 80.5% of MSSA isolates have MIC of vancomycin ≥1. Enterococcus spp. had MIC50 of 1 and MIC90 of 3, whereas coagulase-negative staphylococci had MIC50 of 1 and MIC90 of 2. MIC90 of all the isolates for teicoplanin was between 2 to 3, for linezolid 1.5 to 3 and for daptomucin 0.50 to 0.75.

## Conclusions

A significant creep in the vancomycin MIC for *S. aureus *has occurred in an Indian hospital, which is of important concern as it may lead to treatment failure with vancomycin.
